# Posterior Reversible Encephalopathy Syndrome Presenting as Delirium With Psychosis and Agitation in the Postpartum Period

**DOI:** 10.7759/cureus.56731

**Published:** 2024-03-22

**Authors:** Talitha West, Jason Christopher, Stanislav Arkhipov, Daniel Erickson, Adriana Fitzsimmons

**Affiliations:** 1 Psychiatry, Hackensack Meridian Ocean University Medical Center, Brick, USA; 2 Psychiatry, Hackensack Meridian Jersey Shore University Medical Center, Neptune, USA; 3 Internal Medicine, Hackensack Meridian Jersey Shore University Medical Center, Neptune, USA; 4 Psychiatry, Hackensack Meridian School of Medicine, Nutley, USA

**Keywords:** reproductive psychiatry, obstetrics and gynecology, neurology and critical care, consultation liaison psychiatry, interdisciplinary collaboration, eclampsia, delirium, postpartum psychosis, posterior reversible encephalopathy syndrome (pres)

## Abstract

Posterior reversible encephalopathy syndrome (PRES), which was first described in 1996, is a neurologic condition characterized by a combination of clinical and neuroimaging findings. PRES may arise in the context of preeclampsia, eclampsia, renal failure, and sepsis, among other conditions. Neuropsychiatric symptoms of PRES include altered mental status, agitation, and in some cases psychosis. PRES occurring in the postpartum period is understudied, especially with regard to its psychiatric manifestations. We aim to add to the literature a case of PRES associated with psychosis and agitation in a postpartum woman, highlighting clinical implications and offering suggestions for practice.

A female in her late 20s, with no significant psychiatric or medical history, presented to the hospital at 29 weeks and one day of gestation following a witnessed seizure. She was found to be hypertensive and hyponatremic, was diagnosed with eclampsia, and underwent an emergent cesarean section due to fetal malpresentation. The next day, the patient developed paranoia with acute agitation, and the psychiatry team diagnosed her with delirium with psychosis/agitation secondary to her underlying medical condition. She required intramuscular medications for agitation, was placed in restraints, and was transferred to the ICU for sedation. Subsequently, CT and MRI scans of her head both indicated that she had developed PRES. The patient’s delirium and psychotic behavior resolved after appropriate treatment of her eclampsia.

To our knowledge, this case report is the second documented case in the literature, of a patient who presented with PRES characterized by agitation and psychotic features in the postpartum period. Due to the significant overlap in symptoms between delirium and postpartum psychosis, this case highlights the crucial importance of interdisciplinary collaboration for accurate diagnosis and prompt treatment of PRES in the postpartum period. The case also speaks to the importance of differentiating postpartum psychosis associated with a primary psychiatric disorder from delirium arising in postpartum patients with or without a previous psychiatric history.

## Introduction

Posterior reversible encephalopathy syndrome (PRES) is a syndrome characterized by a combination of clinical and neuroimaging findings. PRES commonly occurs in the context of blood pressure fluctuations, preeclampsia or eclampsia, renal failure, malignancy, autoimmune conditions, and infection or sepsis [1]. Patients with PRES most frequently present with acute onset of encephalopathy, seizures, headaches, and visual symptoms including blurred vision and cortical blindness, nausea, and vomiting [2]. Additional manifestations can include delirium, agitation, and in some cases psychosis [3]. Non-contrast computed tomography (CT) often reveals vasogenic edema. Brain magnetic resonance imaging (MRI), especially the T2-weighted and fluid-attenuated inversion recovery (FLAIR) sequences, is the preferred imaging modality for a diagnosis of PRES. Although patterns consistent with PRES can often be visualized on CT, initial CT findings may be nonspecific, requiring MRI follow-up for confirmation. With the advent of increased use of MRI in the inpatient setting, PRES has become easier to diagnose and treat appropriately [4]. Early recognition of this syndrome is crucial to achieve reversibility and optimize clinical outcomes. Management of PRES often consists of targeting the underlying cause, for example by correcting elevated blood pressure or treating eclampsia [5]. Severe cases of PRES may be life-threatening, requiring aggressive intensive care unit-level supportive care. For example, PRES has been reported in association with acute hemorrhage, obstructive hydrocephalus, and brainstem compression, underscoring the importance of early diagnosis and treatment [6]. Nonetheless, the prognosis of PRES is generally good, and symptom resolution is expected within about one week of onset [7].

In the setting of agitation and psychosis in the postpartum period, it is important (although sometimes difficult) to differentiate between delirium and postpartum psychosis (PP). Despite not being classified as a separate diagnostic entity in the Diagnostic and Statistical Manual of Mental Disorders, PP is acknowledged via a specifier (“with postpartum onset”) as part of a primary psychiatric disorder such as brief psychotic disorder, major depressive disorder, or bipolar disorder [8]. Like other primary psychiatric disorders, PP is frequently characterized by auditory hallucinations [9]. By contrast, auditory hallucinations are relatively rare in delirium, with visual hallucinations being the most common type of hallucination affecting delirious patients [10]. Previous authors have described postpartum psychosis as a primary psychiatric disorder with an atypical phenotype, in that patients with PP often experience delirium-like symptoms, depersonalization, visual and other non-auditory (e.g., tactile) hallucinations, and bizarre delusions or delusions relating to the child or childbirth experience [9]. In clinical practice, these atypical features of PP can present challenges when attempting to differentiate PP from delirium. PP typically occurs within the initial two weeks postpartum and presents with confusion, delusions, and a waxing and waning consciousness similar to that of delirium. PP can also present with manic, depressive, or mixed manic and depressive symptoms [11].

Risk factors for PP include preeclampsia, eclampsia, peripartum infections, cesarean section, and a personal or family history of bipolar disorder [12,13]. Although rare in the general population, PP is significantly more common among women with a personal history of bipolar disorder. An estimated 25% of mothers with bipolar disorder will experience postpartum psychosis, with symptom onset usually occurring in the first two weeks following delivery [13]. Treatment approaches for PP have relied on the use of antipsychotics, mood stabilizers, hormone therapy, propranolol, and electroconvulsive therapy (ECT). Some evidence of efficacy exists for all these approaches, including hormone therapy when provided in the context of documented estradiol deficiency [14]. Most studies assessing treatment of PP have included fewer than 10 patients, however a handful of relatively larger cohort studies appear in the literature. At present, the treatments with the strongest evidence base are lithium, antipsychotics, and ECT. Of note, a relatively large (n=64) study provided support for the use of lithium in both acute and maintenance treatment [15], and smaller studies have found evidence for the use of ECT to induce remission [16].

As detailed above, PRES has been associated with varied neuropsychiatric manifestations including encephalopathy, agitation, and sometimes psychosis. Conditions affecting peripartum women, including preeclampsia and eclampsia, can contribute to the development of PRES. However, PRES occurring in the postpartum period remains understudied, especially with regard to its psychiatric symptoms. Our aim is to add to the literature a case of PRES associated with psychosis and agitation in a postpartum woman, discussing clinical implications and offering suggestions for practice.

This article was presented as a conference poster at the 2023 American Psychiatric Association Annual Meeting on May 20, 2023.

## Case presentation

We reviewed a G3P0020 in her late 20s, with no significant past medical history aside from two miscarriages, who presented to our emergency department at 29 weeks and one day of gestation following a witnessed seizure. According to her husband, the patient was in the car when she suddenly turned to one side, became stiff, and was unresponsive for about a minute. The patient did not recall any of the events surrounding her seizure, however she did note that for the previous two weeks, she had been experiencing headaches and edema of her hands and feet bilaterally. In the emergency department, the patient’s blood pressure was 161/99 mmHg, and she received labetalol 20 mg via IV push, and a 4 g bolus of magnesium sulfate. She was admitted to the labor and delivery service with a diagnosis of eclampsia. On admission, she was found to be hyponatremic with serum sodium of 134 mmol/L. Bedside ultrasound revealed the fetus to be in a transverse lie. Due to eclampsia and fetal malpresentation, she underwent an emergent cesarean section without complications. On the morning of hospital day two, the obstetric team evaluated the patient at follow-up and documented that she was recovering well with pain management (ibuprofen and oxycodone acetaminophen) and magnesium sulfate infusion at 2 g per hour. However, her hyponatremia had worsened to 127 mmol/L, and the perinatologist prescribed furosemide 20 mg daily.

In the evening on hospital day two, nursing staff called a rapid response for the patient due to agitation, and a repeat metabolic panel indicated that her hyponatremia had again worsened to 125 mmol/L. Nurses observed the patient standing in the hallway outside her room, arguing loudly with her husband and hospital staff. The patient screamed obscenities, pulled out her IV line, and demanded to leave the hospital so that she could take care of her dogs. She demonstrated profound confusion and impaired attention and was disoriented to time and situation, stating that she had no memory of delivering a baby and that she had already been in the hospital for over one week. The internal medicine team evaluated the patient and consulted psychiatry due to her acute behavioral change and for assistance in determining her capacity to leave the hospital against medical advice (AMA). Internal medicine also consulted nephrology, requesting a full hyponatremia workup, and recommended a CT scan of the patient's head once she became able to tolerate this. The patient remained agitated despite attempts at verbal de-escalation.

The psychiatry team evaluated the patient, determining that she lacked the capacity to leave the hospital AMA, as she was unable to appreciate the risks of her condition. During her initial mental status exam, the patient was uncooperative and appeared guarded and hostile. She exhibited pressured and disorganized speech. Her affect was labile and expansive, and her thought process was tangential. Thought content included paranoid ideation about hospital staff. Memory, insight, judgment, and impulse control were all poor. Psychiatry ordered as-needed medications for agitation, including haloperidol 2 mg (oral or intramuscular), lorazepam 2 mg (oral or intramuscular), and diphenhydramine 50 mg (oral or intramuscular). Psychiatry also recommended consulting a neurologist due to the patient's abrupt change in mental status. The patient reluctantly agreed to take 2 mg oral haloperidol, however her agitation remained unresolved. Therefore, she received an additional 2 mg of intramuscular haloperidol, along with intramuscular lorazepam and diphenhydramine. The patient's blood pressure remained in the severe range (170-180/100s), however she refused a 10 mg dose of nifedipine. Her agitation did not resolve after receiving haloperidol, lorazepam, and diphenhydramine, therefore she was placed in restraints to prevent further disruptions to her medical treatment. The obstetric team consulted the critical care team, who agreed to transfer the patient to the intensive care unit (ICU) for sedation and further management of her eclampsia.

Early in the morning on hospital day three, the patient was transferred to the ICU, where she received sedation with dexmedetomidine and blood pressure control via clevidipine drip. The ICU team consulted neurology, who ordered an electroencephalogram (EEG) and a non-contrast CT scan of the patient's head, which indicated “subtle areas of decreased attenuation” in the bilateral occipital lobes, consistent with PRES. The patient’s EEG revealed no epileptiform discharges, therefore neurology decided to defer initiating antiepileptic medications and recommended performing an MRI scan of her head following weaning from sedation. On day three, the patient's sodium level improved to 130 mmol/L and her blood pressure began to normalize. Nephrology evaluated the patient, determined that her hyponatremia was "multifactorial likely hypervolemic hyponatremia in setting of anasarca," discontinued furosemide, and placed her on fluid restriction. The psychiatry team followed up with the patient in the ICU, however, she was still sedated with dexmedetomidine. Psychiatry obtained collateral from the patient's husband and mother, who both confirmed that she had no personal or family psychiatric history. They also reported that at baseline, she was independent in all activities of daily living and that her agitated behavior represented a gross departure from her usual conduct. The psychiatry team diagnosed the patient with delirium due to her underlying medical condition. Psychiatry did not suspect a primary psychiatric disorder given the acute mental status change she experienced in the setting of her underlying medical condition. An electrocardiogram performed on day three indicated that the patient's QT interval, corrected for heart rate (QTc) was 503, therefore psychiatry recommended holding antipsychotics and using as-needed lorazepam for any further episodes of acute agitation.

**Figure 1:**
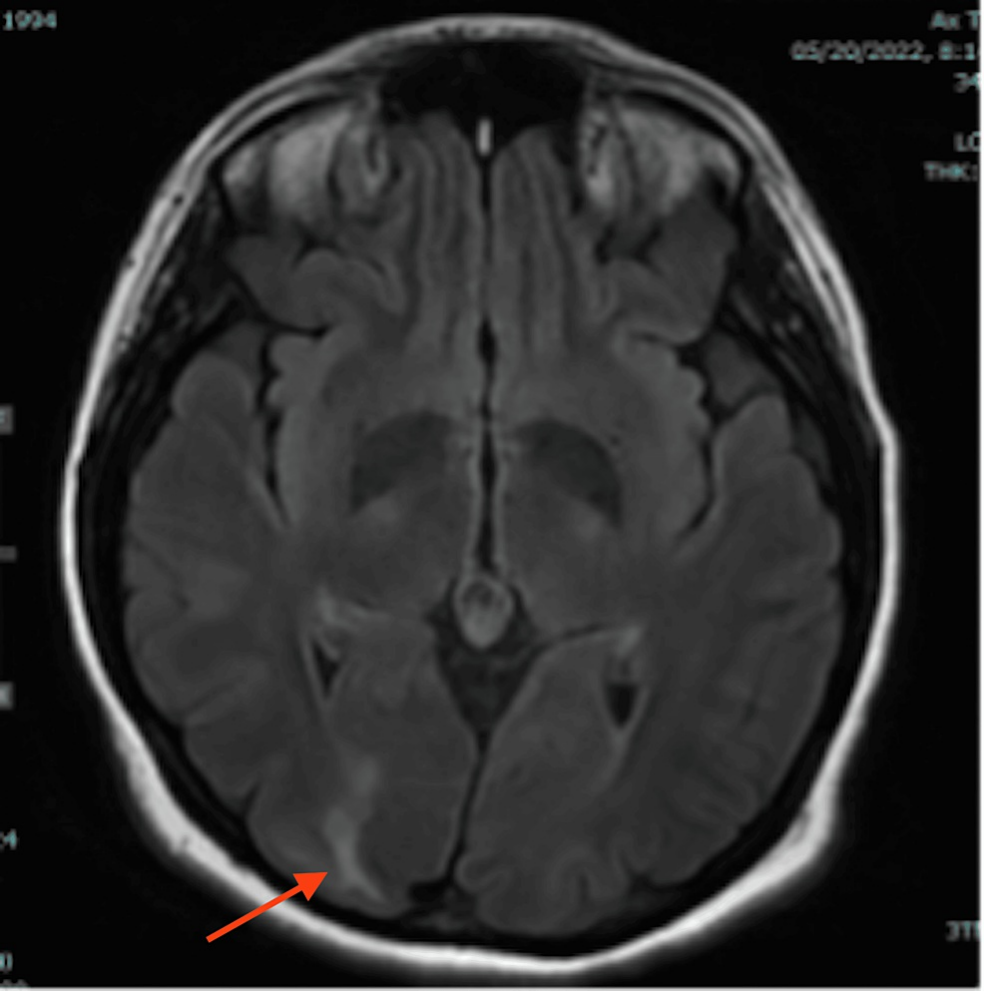
T2/FLAIR MRI of the brain (image I) FLAIR: Fluid attenuated inversion recovery The red arrow shows hyperintensity in the right occipital lobe.

**Figure 2:**
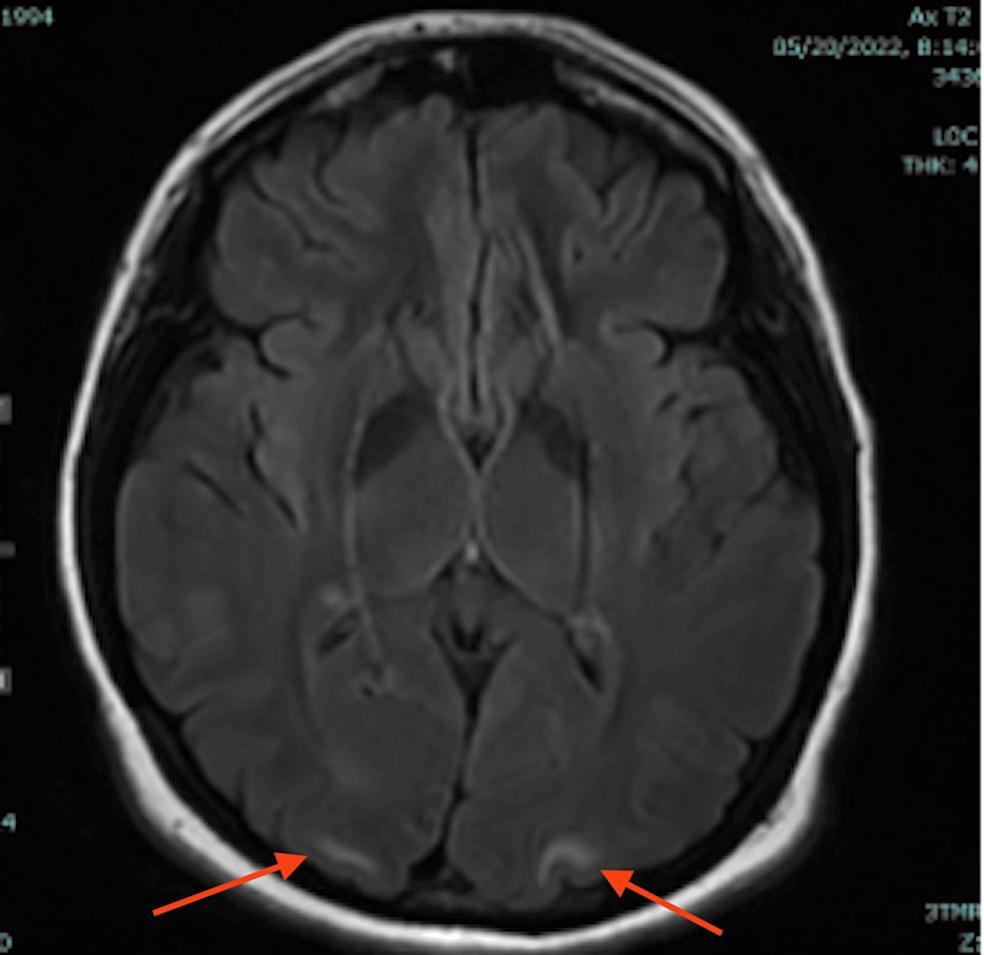
T2/FLAIR MRI of the brain (image II) FLAIR: Fluid attenuated inversion recovery Red arrows show hyperintensities in bilateral occipital lobes.

**Figure 3:**
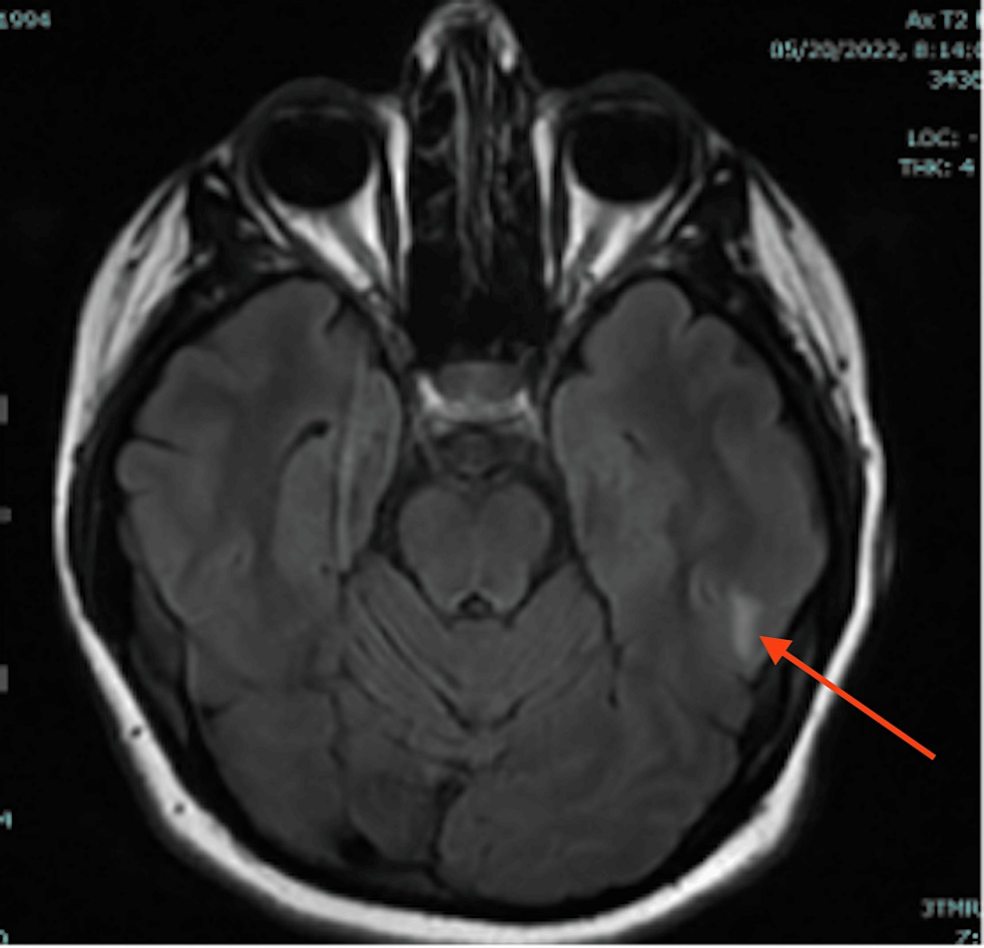
T2/FLAIR MRI of the brain (image III) FLAIR: Fluid attenuated inversion recovery The red arrow shows the hyperintensity in the left temporal lobe,

On hospital day four, the ICU team weaned the patient from dexmedetomidine sedation. Her behavioral disturbance had resolved, and she was no longer agitated. However, at psychiatric follow-up, she remained confused and was still unable to recall the events surrounding her admission. Her hyponatremia improved to 138 mmol/L with fluid restriction, and her blood pressure continued to improve (127-157/72-93). As she was now able to tolerate oral medications, she was transitioned to labetalol 400 mg every eight hours and nifedipine 30 mg daily for blood pressure control. Neurology ordered an MRI of her head without contrast, which showed areas of increased T2/FLAIR signal in the subcortical white matter of the bilateral occipital lobes (Figures 1, 2), and left temporal lobe (Figure 3), consistent with PRES. 

By hospital day five, the patient displayed no residual evidence of delirium and was alert and oriented, with a full affect, stable mood and behavior, and no signs of psychosis. The patient continued to endorse amnesia for the events surrounding her admission (including a memory loss of three to four days) but otherwise was feeling well. With her delirium, hypertension, and hyponatremia resolved, the patient was deemed stable for discharge on hospital day six. The neurology team discussed the possibility of starting an antiepileptic drug, however it was decided that this was not necessary due to her normal EEG and the fact that she experienced only one seizure in the context of eclampsia. The patient was scheduled for appropriate outpatient follow-up appointments and discharged home. At her last contact with the psychiatry team about 16 months following her initial presentation, she remained psychiatrically asymptomatic and had experienced no further seizures.

## Discussion

To our knowledge, the literature contains only one previous case report documenting PRES characterized by agitation and psychotic features in the postpartum period. That case described an 18-year-old Colombian woman with no prior psychiatric history, who developed a holocranial headache and a generalized tonic-clonic seizure in the context of postpartum eclampsia. Her neuropsychiatric symptoms resolved rapidly and completely following the administration of diazepam, magnesium sulfate, phenytoin, labetalol, and oral antihypertensives [17]. The present article adds to the literature an additional case in which PRES co-occurred with psychosis in the postpartum period. Like the patient in the previous case report, our patient had no prior history of psychiatric illness. Unlike the previous case, in which the patient had an entirely normal pregnancy (including normal blood pressure throughout pregnancy) until her membranes ruptured prematurely and her fetus presented in breech at about 39 weeks gestation, our patient was diagnosed with eclampsia at around 29 weeks gestation following the generalized tonic-clonic seizure that led to her presentation at our hospital. By contrast, the patient in the previous case report developed postpartum eclampsia with a generalized tonic-clonic seizure occurring 14 hours after cesarean delivery.

The current case highlights the crucial importance of interdisciplinary collaboration in patients with suspected PRES, which several previous writers on this topic have recognized. In our patient’s case, although she was initially admitted under the obstetrics service, she promptly underwent evaluation by members of the perinatology, nephrology, critical care, neurology, and psychiatry teams. This timely collaboration between specialties facilitated the prompt diagnosis of PRES, and the patient's neuropsychiatric symptoms resolved within a few days of onset. As our case illustrates, the onset of PRES tends to be acute, and its generally favorable prognosis depends on early diagnosis and treatment of underlying causes, comorbidities, and sequelae (e.g., eclampsia, hyponatremia, and psychosis). Hence, practitioners faced with a potential case of PRES should err on the side of seeking early consultations with specialists rather than waiting for diagnostic clarification.

We concur with the authors of the previous Colombian case report that early identification of PRES is of utmost importance in pregnant and postpartum patients with concomitant preeclampsia or eclampsia [17]. This is because both PRES and eclampsia can cause severe neurological complications and because the combined impact of these two conditions on symptom progression is still poorly understood. Accordingly, physicians should strive for prompt diagnosis and treatment of patients with suspected PRES occurring in the context of eclampsia spectrum conditions, as this may avert irreversible neurological disability. Following the resolution of a patient's acute symptoms, practitioners should be prepared to discuss with postpartum patients the risks and benefits of antiepileptic maintenance therapy, including potential teratogenic effects on future pregnancies. Although our neurology team determined that anticonvulsant medications were not required in the current case, patients with PRES often do receive antiepileptic therapy. Due to the emerging nature of PRES, there are no general practice guidelines or recommendations regarding which specific drugs to use or the ideal duration of antiepileptic therapy. However, the current consensus seems to be that it is safe to taper antiepileptic drugs once a patient has become asymptomatic and imaging findings have returned to normal [18].

Finally, the present case has important implications for psychiatric practice in the consultation-liaison setting. A recent case series conducted in the liaison neuropsychiatry service of a large teaching hospital in London identified 47 patients who presented with PRES between April 2010 and April 2019, many of whom required psychiatric services either prior to or during the admission in which they were diagnosed with PRES [3]. In this study, a significant minority of patients with PRES developed altered mental status during hospitalization but were never seen by a psychiatrist. According to the authors, 26% of cases developed either mental status change or psychiatric symptoms during admission, including speech disturbance, confusion, agitation, hallucinations, disinhibition, low mood, delusions, unpleasant or vivid dreams, religious preoccupation, self-harm, or anxiety. These data, along with the Colombian case study and our own, contribute to a growing body of evidence suggesting that PRES often provokes altered mental status and sometimes precipitates psychotic symptoms, regardless of whether a patient has any past psychiatric history. This point underscores the urgent importance of the early involvement of a consult liaison psychiatrist in cases where PRES presents with altered mentation. Prompt involvement of psychiatry is indispensable not only for appropriate treatment of psychiatric symptoms and accurate diagnosis of delirium but also to ensure adequate psychiatric follow-up post-hospitalization if necessary.

## Conclusions

This case report underscores the benefits of prompt interdisciplinary collaboration in cases of PRES. In our case, collaboration between the obstetrics, perinatology, nephrology, critical care, neurology, and psychiatry teams resulted in an optimal clinical outcome, as our patient experienced a complete and sustained recovery after receiving appropriate psychiatric and medical care.

The case also serves as a reminder of the paramount clinical importance of correctly differentiating primary postpartum psychosis from delirium to ensure timely medical care for a delirious patient’s underlying medical condition (in our case, eclampsia). Psychiatrists and other physicians previously unfamiliar with PRES should add it to the already broad differential diagnosis of organic conditions that can provoke acute delirium or psychosis. At present, the state of medical knowledge about PRES is still meager. Larger studies are needed to elucidate the rate at which PRES co-occurs with psychiatric symptoms and to determine whether the effects of PRES on mental health are generally transient or persistent.
